# Transcriptomic Approaches to Investigate the Anti-Aging Effects of Blueberry Anthocyanins in a Caenorhabditis Elegans Aging Model

**DOI:** 10.3390/antiox14010035

**Published:** 2024-12-30

**Authors:** Jie Ding, Jiahui Liu, Qingqi Guo, Na Zhang

**Affiliations:** 1 College of Life Science, Northeast Forestry University, Harbin 150040, China; dingjie@nefu.edu.cn (J.D.); 2022120285@nefu.edu.cn (J.L.); 2 College of Food Engineering, Harbin University of Commerce, Harbin 150028, China

**Keywords:** blueberry anthocyanins, caenorhabditis elegans, transcriptomics, anti-aging

## Abstract

This study investigates the anti-aging effects of various concentrations of blueberry anthocyanins (BA) on the lifespan and health-related phenotypes of Caenorhabditis elegans. Blueberry anthocyanins were administered at concentrations of 50.0 μg/mL, 200.0 μg/mL, and 500.0 μg/mL, and their effects on nematode lifespan, locomotion, pharyngeal pumping rate, and the accumulation of lipofuscin and reactive oxygen species (ROS) were examined. Transcriptomic analysis was conducted to explore the regulatory effects of BA on anti-aging molecular pathways and key genes in *C. elegans*. Results showed a significant, dose-dependent extension of lifespan, improvement in locomotion and pharyngeal pumping rate, and reduction in lipofuscin and ROS accumulation. Transcriptomic analysis revealed that BA activated anti-aging pathways such as FOXO, IIS, and PI3K/Akt, upregulating critical genes like *daf-16*. These findings highlight the potential of blueberry anthocyanins as promising anti-aging agents through multiple physiological and molecular mechanisms.

## 1. Introduction

Aging is a complex biological process characterized by the gradual decline in cellular function, influenced by environmental factors such as oxidative stress and genetic factors [1,2,3]. Key mechanisms of ageing include telomere shortening, cellular senescence, and the accumulation of reactive oxygen species (ROS). Recent reviews have highlighted the roles of genetic regulation, epigenetic changes, and the involvement of inflammatory pathways in the ageing process [4]. The role of oxidative damage in aging has made the study of natural antioxidants a major focus in the search for therapeutic strategies to combat age-related decline. Blueberry anthocyanins (BA), natural polyphenolic compounds with strong antioxidant and anti-inflammatory properties, have garnered significant attention for their potential to delay aging and mitigate age-related conditions [5,6]. BA are known to improve blood circulation, enhance antioxidant defenses, and protect against oxidative damage, making them promising candidates for anti-aging interventions [7].

*C. elegans*, a non-parasitic nematode, is widely used as a model organism in aging research owing to its short lifespan and genetic similarities to humans [8]. The transparency of *C. elegans* also facilitates direct observation of physiological changes. Transcriptomic analysis, which involves studying RNA expression to understand gene regulation, provides detailed insights into the molecular mechanisms affected by interventions such as anthocyanins [9]. While *C. elegans* has been invaluable in aging studies, recent research also highlights the relevance of mammalian models in understanding the broader implications of aging and potential therapeutic interventions. Studies in mammals have shown similar pathways activated by anthocyanins, including the FOXO signaling pathway, which plays a key role in aging and stress resistance [10,11]. This study aims to elucidate the anti-aging mechanisms of BA through transcriptomic analysis, focusing on changes in gene expression and the biological pathways impacted in *C. elegans* [12,13,14]. Understanding these interactions may pave the way for utilizing BA as therapeutic agents against aging and age-related conditions.

Current research on BA (blueberry anthocyanins) has started to illuminate their potential anti-aging effects on Caenorhabditis elegans. Previous studies, such as those by Gonzales, Zhao, and Nas [15,16,17] have demonstrated that anthocyanins can enhance lifespan and vitality in *C. elegans*. These findings highlight the role of anthocyanins in promoting stress resistance and delaying aging.

However, a detailed understanding of the molecular mechanisms underlying these effects remains underexplored [18]. In particular, the pathways and gene regulatory networks influenced by anthocyanins have not been fully elucidated. This study aims to delve into these mechanisms using transcriptomic analysis to identify changes in gene expression and the biological pathways affected by BA. By focusing on key aging-related pathways, such as FOXO, IIS, and PI3K/Akt [19]. This study aims to elucidate the anti-aging mechanisms of BA through transcriptomic analysis, focusing on the key genes and pathways affected in *C. elegans*. By identifying how BA modulate these pathways, this research deepens our understanding of their therapeutic potential and provides reference and guidance for further studies in higher organisms, including humans.

## 2. Materials and Methods

### 2.1. Materials and Reagents

BA (secondary purified) were obtained from the Key Laboratory of Forest Food Resources Utilization in Heilongjiang Province. The secondary purification process involved several steps. First, the anthocyanins were selectively concentrated using macroporous resin adsorption, a method that utilizes the selective adsorption properties of the macroporous resin to concentrate the anthocyanins from the raw extract, effectively separating them from other impurities. After the initial concentration, the anthocyanins were eluted using an ethanol-water solution, further removing non-target components and improving the purity of the anthocyanins. Next, gel filtration chromatography was used to further purify the anthocyanins. This method separates anthocyanins from residual impurities based on molecular size. Due to the intermediate size of anthocyanin molecules, they passed through the column more efficiently than either larger or smaller impurities, ensuring effective separation. The purified extract was then concentrated under reduced pressure to remove solvents, increasing the concentration of anthocyanins. Finally, the concentrated extract was freeze-dried (lyophilized) to yield a stable powdered form. This process ensured that the anthocyanins remained stable and retained their bioactivity. The extract primarily contains the following bioactive anthocyanin compounds: Cyanidin-3-glucoside, Delphinidin-3-glucoside, Petunidin-3-glucoside, Malvidin-3-glucoside, and Peonidin-3-glucoside. These compounds are the major anthocyanins found in blueberries and are recognized for their significant antioxidant and anti-aging properties. The final product exhibited an anthocyanin purity of 70.18%, suitable for advanced experimental applications. The anthocyanin concentration was measured using the pH differential method, with absorbance measurements taken using a PerkinElmer Lambda 25 UV-Vis Spectrophotometer (PerkinElmer, Waltham, MA, USA). The anthocyanin standards were purchased from Sigma-Aldrich (St. Louis, MI, USA).

The Escherichia coli OP50 strain (uracil-deficient) and wild-type *C. elegans* N2 were preserved in our laboratory. LB broth and agar were procured from Qingdao Hope Bio-Technology Co., Ltd. (Qingdao, China). Disodium phosphate, sodium dihydrogen phosphate, and sodium hypochlorite were sourced from Shanghai Macklin Biochemical Technology Co., Ltd. (Shanghai, China). Cholesterol was obtained from Sangon Biotech (Shanghai) Co., Ltd. (Shanghai, China), and magnesium sulfate from China National Pharmaceutical Group Chemical Reagents Co., Ltd. (Shanghai, China). Additional reagents used in this study, including glucose, chloroform, anhydrous ethanol, isopropanol, anhydrous calcium chloride, magnesium sulfate heptahydrate, sodium hydroxide, sodium chloride, monopotassium phosphate, dipotassium phosphate, citric acid monohydrate, potassium citrate monohydrate, ethylenediaminetetraacetic acid (EDTA), ferrous sulfate heptahydrate, manganese chloride tetrahydrate, zinc sulfate heptahydrate, copper sulfate pentahydrate, disodium phosphate dodecahydrate, cholesterol, and sodium hypochlorite, were also obtained from China National Pharmaceutical Group Chemical Reagents Co., Ltd. (China). 5-Fluoro-2′-deoxyuridine was purchased from Sigma-Aldrich (USA). TRIzol reagent and SuperReal PreMix Plus (SYBR Green) were sourced from Tiangen Biotech Co., Ltd. (Beijing, China), while the reverse transcription kit was obtained from Vazyme Biotech Co., Ltd. (San Diego, CA, USA). All chemical reagents utilized were of analytical grade.

### 2.2. Instruments and Equipment

The TA DHR-2 autoclave was supplied by Shanghai Lichen Technology Co., Ltd. (Shanghai, China). The SW-CJ-1 clean bench was used in the experiments. The DM500 microscope was purchased from Leica Microsystems (Tokyo, Japan), and the JC-QX-1 constant temperature incubator was sourced from Qingdao JuChuang Environmental Protection Equipment Co., Ltd. (Qingdao, China). The LSM900 confocal laser scanning microscope was provided by Zeiss (Jena, Germany). The MY-10 handheld grinder was purchased from Shanghai Jingxin Industrial Development Co., Ltd. (Shanghai, China). The refrigerated centrifuge was supplied by Eppendorf (Hamburg, Germany), and the QuantStudio 3.0 real-time fluorescence quantitative polymerase chain reaction (PCR) system was provided by ABI (Los Angeles, CA, USA).

### 2.3. C. elegans Culture Methods

#### 2.3.1. Preparation of Solid Medium Containing BA

Nematode growth medium (NGM) was prepared by dissolving 1.5 g of NaCl, 1.25 g of peptone, and 8.5 g of agar in distilled water, followed by sterilization at 121 °C for 20 min. After sterilization, 0.5 mL each of 1 mol/L CaCl_2_, 1 mol/L MgSO_4_, 5 mg/mL cholesterol ethanol solution, and 12.5 mL of 1 mol/L PBS buffer were added and thoroughly mixed. BA were dissolved in PBS, filtered for sterility, and mixed with *E. coli* OP50 in a 1:1 ratio. The final concentrations of BA in the nematode culture medium were 50, 200, and 500 μg/mL. The mixture was applied to prepared plates and stored at 4 °C until use. To prevent excessive offspring, mNGM medium was prepared by adding 50 μL of 2.5% 5-Fluoro-2′-deoxyuridine (5-FUdR) solution per 100 mL of NGM after sterilization and cooling.

#### 2.3.2. Bacterial Activation and *C. elegans* Culture

The *E. coli* OP50 strain was streaked on plates, and single colonies were picked and cultured at 37 °C, 180 rpm, until reaching the logarithmic growth phase (OD_600_ = 0.6). Cultures were stored at 4 °C until use. *C. elegans* were cultured on plates containing OP50 or a mixture of OP50 and BA (as described in the previous section) at 20 °C. Plates were replaced every two days, and *C. elegans* were transferred by cutting a small section of the plate.

#### 2.3.3. Synchronization of *C. elegans*

A 2 mol/L NaOH solution was prepared and mixed with 10% NaClO in a 1:2 ratio to create a *C. elegans* lysis solution (prepared immediately prior to use). Following the method of [20], synchronization of *C. elegans* was performed. The *C. elegans* were washed off the plates using the lysis solution and collected in centrifuge tubes. After vortexing for 5–8 min, the *C. elegans* were washed three times with M9 buffer, and the eggs were incubated at 20 °C for 72 h until they developed to the L4 larval stage, which represents the fourth and final larval stage before adulthood, thereby completing synchronization. This ensured that the population was uniformly staged for subsequent experimental procedures.

#### 2.3.4. Grouping of *C. elegans* for Experiments

Experimental plates were divided into the following groups: Control group: Plates coated with 100 μL of OP50 culture. Treatment groups: Plates coated with 100 μL of a mixture of OP50 and BA at final concentrations of 50.0, 200.0, and 500.0 μg/mL.

### 2.4. Nematode Assays

#### 2.4.1. Experimental Evaluation of BA Impact on *E. coli* OP50

A total of 200 μL of *E. coli* OP50 was added to 96-well plates and mixed with 75 μL of different concentrations of BA (0.0–1000.0 μg/mL), then incubated at 37 °C. After 24 h, the optical density (OD) at 600 nm was measured to evaluate the effect of BA on *E. coli* OP50 growth.

#### 2.4.2. Assessment of BA Impact on the Lifespan of Wild-Type *C. elegans*

The lifespan of wild-type *C. elegans* was assessed using NGM plates coated with *E. coli* OP50 and BA at final concentrations of 0, 50.0, 200.0, and 500.0 μg/mL. Plates also contained 5-FUdR to inhibit reproduction. Forty synchronized L4-stage *C. elegans* were transferred to the NGM plates and incubated at 20 °C, marking day 0 of the lifespan experiment. Survival was monitored daily, and nematodes were transferred to fresh plates every two days. Death was determined by the absence of response to gentle prodding with a *C. elegans* picker. Nematodes that crawled off the plate or desiccated were excluded from the analysis [21].

#### 2.4.3. Assessment of BA Impact on the Body Bending Frequency of *C. elegans*

Synchronized L4-stage *C. elegans* were transferred to plates coated with *E. coli* OP50 and BA at concentrations of 0.0, 50.0, 200.0, and 500.0 μg/mL and fed for 5 days. After acclimating for 30 s, the body bends of individual nematodes were observed and recorded for 30 s. Fifteen *C. elegans* per group were tested.

#### 2.4.4. Assessment of BA Impact on Pharyngeal Pumping Frequency of *C. elegans*

Synchronized L4-stage *C. elegans* were transferred to plates coated with *E. coli* OP50 and BA at concentrations of 0.0, 50.0, 200.0, and 500.0 μg/mL and fed for 5 days. After acclimating for 30 s, the pharyngeal pumping frequency of individual nematodes was observed and recorded for 30 s. Fifteen *C. elegans* per group were tested.

#### 2.4.5. Assessment of BA Impact on Reproductive Capacity of *C. elegans*

After synchronization, *C. elegans* eggs were incubated at 20 °C until they developed into adults. The adults were then transferred to NGM plates containing BA at concentrations of 0.0, 50.0, 200.0, and 500.0 μg/mL (one *C. elegans* per plate). Each day, the adults were transferred to a fresh plate, and the number of offspring was counted after they developed into larvae. The experiment was independently repeated three times, and the data were plotted using GraphPad Prism 8 software.

#### 2.4.6. Assessment of BA Impact on Lipofuscin Accumulation in *C. elegans*

Synchronized L4-stage *C. elegans* were transferred to plates coated with *E. coli* OP50 and BA at concentrations of 0.0, 50.0, 200.0, and 500.0 μg/mL and incubated for 5 days at 20 °C. The *C. elegans* were then placed on agarose pads containing 2.0 mmol/L levamisole and anesthetized for 1 min. Lipofuscin autofluorescence was observed under an inverted fluorescence microscope with excitation at 380 nm and emission at 430 nm. Images were captured under identical exposure conditions, and ImageJ software (version 1.53v) was used to quantify fluorescence intensity [22].

#### 2.4.7. Assessment of BA Impact on ROS Accumulation in *C. elegans*

Synchronized L4-stage *C. elegans* were transferred to plates coated with *E. coli* OP50 and BA at concentrations of 0.0, 50.0, 200.0, and 500.0 μg/mL and incubated for 5 days at 20 °C. The *C. elegans* were washed off the plates with M9 buffer and collected in Eppendorf tubes. DCFH-DA fluorescent probe was added to the tubes at a final concentration of 50.0 μmol/L, and the *C. elegans* were incubated at 20 °C in the dark for 30 min. Fluorescence was observed under an inverted fluorescence microscope, and images were captured under consistent conditions. Fluorescence intensity was quantified using ImageJ software [23].

### 2.5. Transcriptome Sequencing of C. elegans

#### 2.5.1. Sample Collection

*C. elegans* were cultured as described above. On the third day of adulthood, *C. elegans* from each treatment group were collected, washed three times with M9 buffer to remove bacteria, ground on ice, and centrifuged at 10,000 rpm for 5 min at 4 °C to obtain supernatants.

#### 2.5.2. Total RNA Extraction

Total RNA was extracted using TRIzol reagent following the manufacturer’s protocol. RNA samples were stored in liquid nitrogen and later used for transcriptome sequencing.

#### 2.5.3. RNA Library Construction

Before constructing the RNA library, RNA quality was assessed to ensure that the samples were suitable for library preparation. For each sample, 5 μg of total RNA was used for library construction. Eukaryotic mRNA was enriched using oligo(dT) magnetic beads, and the mRNA was then fragmented using fragmentation buffer. The fragmented mRNA was used as a template for synthesizing the first-strand cDNA with random hexamer primers, followed by second-strand cDNA synthesis. The cDNA was purified with magnetic beads, amplified by PCR, and the quality of the library was assessed before sequencing.

#### 2.5.4. Genomic Analysis

SeqPrep software (version 1.3) was used to filter the raw sequencing data and obtain high-quality reads. The sequencing data were aligned to the reference genome (WBcel235, source: http://metazoa.ensembl.org/Caenorhabditis_elegans/Info/Index (accessed on 20 March 2024). Gene expression was quantified, and differentially expressed genes were subjected to GO and KEGG functional analyses.

### 2.6. Validation of Differentially Expressed Genes by qRT-PCR

#### 2.6.1. Reverse Transcription of cDNA

The RNA obtained from Section 2.5.2 was diluted to approximately 300 ng/μL. The reaction system for gDNA removal was prepared as follows: 2 μL of 5× gDNA Eraser Buffer, 1 μL of gDNA Eraser, and 7 μL of RNA. The mixture was incubated at 42 °C for 2 min. The reverse transcription reaction was then set up according to the reverse transcription kit’s protocol and incubated at 37 °C for 15 min, followed by 85 °C for 5 s. The resulting cDNA was stored at −80 °C.

#### 2.6.2. qRT-PCR Validation

Real-time PCR was performed using a 10 μL reaction system containing 5 μL of 2× qPCR Mix, 0.4 μL of forward primer, 0.4 μL of reverse primer, 0.5 μL of cDNA, and 3.7 μL of ddH_2_O. The reaction was carried out on a fluorescence quantitative PCR machine with the following cycling conditions: 95 °C for 30 s, followed by 40 cycles of 95 °C for 5 s, 57 °C for 30 s, and 72 °C for 30 s. Fluorescence was collected during the extension phase, and the melting curve ranged from 60 °C to 95 °C with 1 °C increments every 15 s. The 2^−ΔΔCT^ method was used to calculate the relative expression of the target genes. act-1 was used as the reference gene.Results are expressed as fold changes relative to the untreated control group.The genes selected for qRT-PCR validation were chosen based on their significant differential expression identified in the RNA-seq data and their biological relevance to pathways influenced by blueberry anthocyanins (BA), including stress resistance, energy metabolism, and aging regulation. Primer sequences for the *C. elegans* target genes were obtained from the qPrimerDB database (https://biodb.swu.edu.cn/qprimerdb/ (accessed on 5 April 2024). The primers were synthesized by Qingke Biological Technology Co., Ltd. (Wuhan, China), and the sequences were listed in Table 1.

### 2.7. Data Analysis

All experiments were performed in triplicate. Data were analyzed using GraphPad Prism 8.4.3 and SPSS software (version 26). Kaplan-Meier analysis was performed using GraphPad Prism 8.4.3 and SPSS software (version 26), and statistical significance was determined using the log-rank test, *t*-test, and one-way ANOVA. A *p*-value of less than 0.05 was considered to indicate statistical significance.

## 3. Results

### 3.1. Impact of Different Concentrations of BA on E. coli OP50

**Figure 1:**
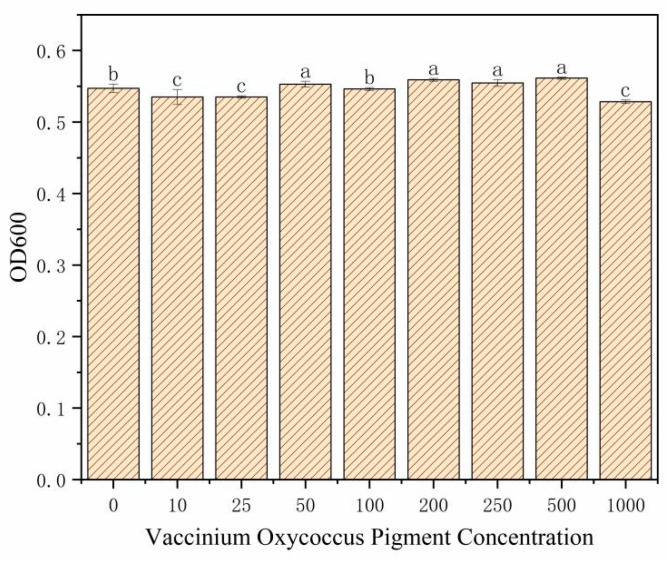
Effects of BA on *E. coli* OP50. Different small letters (a–c) represent significant differences (*p* < 0.05).

As shown in Figure 1, an increase in the concentration of BA results in a slight decrease in the OD600 of *E. coli* OP50. At concentrations below 500.0 μg/mL, there were no significant differences in the OD600 values compared to those of normally growing *E. coli* OP50 (OD600 = 0.54 ± 0.01). However, at a concentration of 1000.0 μg/mL, the OD600 value significantly dropped to 0.52 ± 0.01, indicating inhibited bacterial growth compared to the control group. Therefore, at treatment concentrations below 500.0 μg/mL, BA do not significantly affect the growth of *E. coli* OP50 and, by extension, do not adversely impact the growth of *C. elegans* [24]. Based on these findings, concentrations of 50.0 μg/mL, 200.0 μg/mL, and 500.0 μg/mL of BA were selected for subsequent experiments on *C. elegans*.

### 3.2. Effect of Different Concentrations of BA on the Lifespan of C. elegans

**Figure 2:**
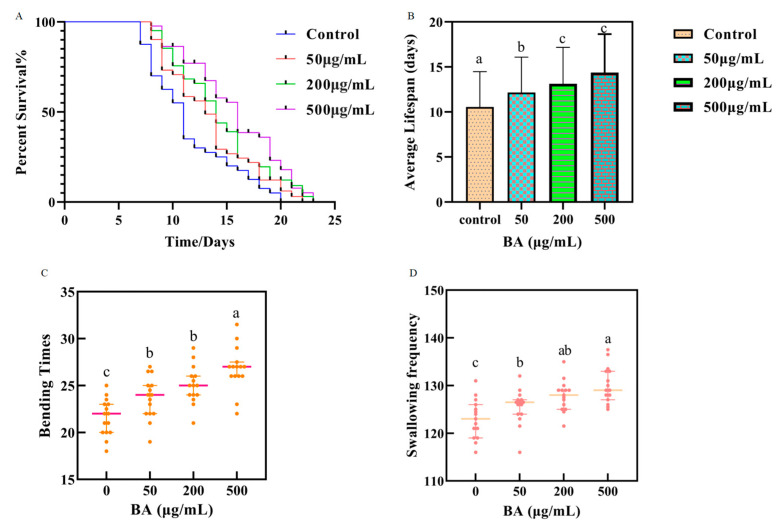
Survival rate of *C. elegans*. (**A**); The effect of BA on the pharyngeal pumping rate of *C. elegans*. (**B**); Mean lifespan of *C. elegans*. (**C**); The effect of BA on the bending frequency of *C. elegans*. (**D**). Different small letters (a–c) represent significant differences (*p* < 0.05). Groups sharing a common letter (e.g., ‘ab’) do not differ significantly from each other, indicating that they are statistically similar.

In this study, the lifespans of 40 wild-type *C. elegans* were assessed. The control group exhibited an average lifespan of 10.55 ± 3.92 days, with a maximum lifespan of 21 days. After treatment with BA, an extension in lifespan was observed. Specifically, at concentrations of 50.0, 200.0, and 500.0 μg/mL BA, as depicted in Figure 2A,C, the average lifespans increased to 12.15 ± 3.94 days, 13.13 ± 4.04 days, and 14.38 ± 4.26 days respectively, which are 1.15, 1.24, and 1.36 times longer than the control group. The maximum lifespans reached 22, 23, and 24 days respectively, showing an extension of 1, 2, and 3 days compared to the aging nematodes.

### 3.3. Impact of Different Concentrations of BA on the Bending Frequency of C. elegans

This study measured the number of bends within 30 s in an M9 buffer solution. As depicted in Figure 2D, compared to the aging nematodes in the control group, those treated with various concentrations of BA demonstrated an increase in bending frequency.

Specifically, nematodes treated with 50.0 μg/mL of BA exhibited a slight increase in bends per unit time, averaging 23.67 ± 2.15, compared to the control group’s 21.60 ± 1.90, although this difference was not statistically significant. Treatment with 200.0 μg/mL and 500.0 μg/mL resulted in bending frequencies of 25.17 ± 1.94 and 26.80 ± 2.28, respectively, showing no significant differences between these two concentrations. However, when compared to the control group, the low, medium, and high concentrations of BA resulted in bending frequency increases of 1.10%, 8.50%, and 12.40%, respectively. These results indicate that BA can alleviate the decline in locomotor ability associated with aging and that its effects are dose-dependent within the concentrations tested in this experiment.

### 3.4. Impact of Different Concentrations of BA on the Pharyngeal Pumping Frequency of C. elegans

Our experimental results (as shown in Figure 2B) indicate that, after five days of treatment, the groups administered with drugs demonstrated a higher pumping rate compared to the control. Specifically, *C. elegans* treated with 50.0 μg/mL BA showed a pumping frequency of 125.53 ± 3.52, slightly higher than the control group at 122.97 ± 3.97. At 200.0 μg/mL and 500.0 μg/mL BA, the frequencies were 127.8 ± 3.12 and 130.13 ± 3.74, respectively. These results suggest that BA enhances the feeding capacity of the nematodes, and the lifespan extension observed is not induced by dietary restriction, aligning with findings by Edwards [25]. This demonstrates that BA improve pharyngeal pumping efficiency in *C. elegans*, contributing to their overall health and potentially extending their lifespan through mechanisms other than caloric restriction.

### 3.5. Impact of Different Concentrations of BA on the Reproductive Capacity of C. elegans

**Figure 3:**
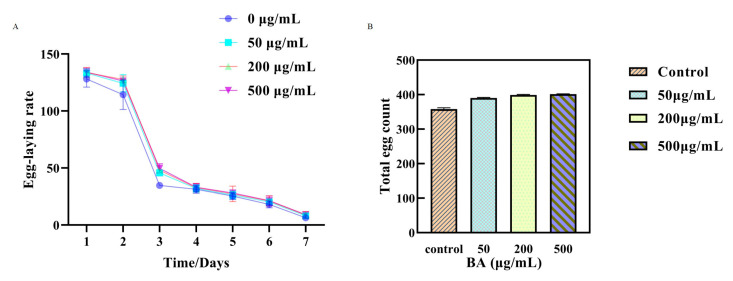
The effect of BA on the reproductive capacity of wild-type *C. elegans*; daily egg-laying rate per *C. elegans* (**A**); total egg-laying count over 7 days (**B**).

The results, as illustrated in Figure 3A,B, showed that there were significant differences in the number of eggs laid by the *C. elegans* in the drug-treated group compared to the control group. Specifically, the nematodes treated with BA laid considerably more eggs on the first, third, fifth, and seventh days of adulthood than those in the control group. These findings suggest that BA can enhance the reproductive capacity of the *C. elegans*, potentially delaying their aging process.

### 3.6. Impact of Different Concentrations of BA on Lipofuscin Accumulation in C. elegans

**Figure 4:**
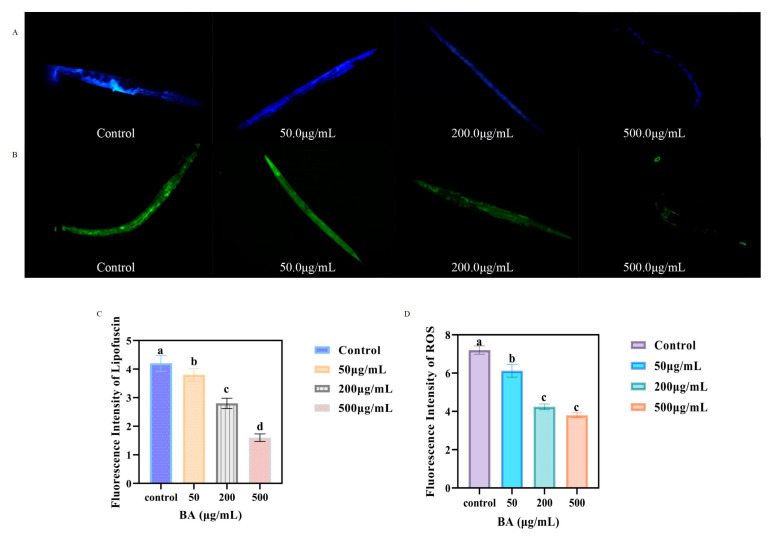
Lipofuscin fluorescence images of *C. elegans* (**A**); ROS fluorescence images of *C. elegans* (**B**); The effect of BA on lipofuscin accumulation in *C. elegans* (**C**); The effect of BA on ROS levels in *C. elegans* (**D**). Different small letters (a–d) represent significant differences (*p* < 0.05).

In this study, we utilized an inverted fluorescence microscope to capture images of lipofuscin fluorescence within *C. elegans* and assessed the fluorescence intensity using Image J software. As shown in Figure 4A, *C. elegans* in the control group (aged *C. elegans*) and those treated with 50.0 μg/mL BA displayed strong blue fluorescence. Conversely, *C. elegans* treated with 200.0 μg/mL and 500.0 μg/mL BA exhibited dimmer blue fluorescence. As the concentration of BA increased, the fluorescence intensity progressively decreased, showing significant differences between the concentrations.

### 3.7. Impact of Different Concentrations of BA on ROS Levels in C. elegans

Fluorescence microscopy images (Figure 4B) showed that the control group of *C. elegans* exhibited strong green fluorescence, indicating higher ROS levels associated with natural aging and confirming that natural aging correlates with increased endogenous ROS levels.

Post-treatment with varying concentrations of BA, the naturally aged *C. elegans* displayed weakened green fluorescence, with the 500.0 μg/mL BA treatment group showing the lowest intensity of green fluorescence. This suggests that BA can scavenge ROS, effectively reducing ROS levels in the body, with the reduction being most pronounced at the highest concentration tested.

### 3.8. Anti-Aging Effects of BA on Nematodes

Previous research has shown that adding BA to the diet can extend the lifespan of nematodes, with concentrations of 50.0 μg/mL, 200.0 μg/mL, and 500.0 μg/mL leading to lifespan increases of 1.13, 1.21, and 1.32 times, respectively. Therefore, this experiment opted to use 500.0 μg/mL of BA as the induction dose. Further, studies indicate that after the addition of 500.0 μg/mL of BA, there were improvements in various phenotypes of the nematodes, such as swallowing rate and bending frequency, suggesting that BA slowed the decline of healthy phenotypes in nematodes. Given the effects of BA on nematode lifespan and health phenotypes, transcriptomic sequencing was employed to explore the molecular mechanisms by which BA induce anti-aging effects in nematodes.

### 3.9. RNA Quality Control Results

Using the Illumina high-throughput sequencing platform, 10 samples were analyzed, including 5 control and 5 treated with BA, with each sample generating an average of 7.21 Gb of data. As shown in Table 2, all ten samples had OD 260 nm/280 nm ratios above 1.80, indicating no contamination with proteins or other impurities. The OD 260 nm/230 nm ratios ranged from 2.1 to 2.6, suggesting the absence of organic reagent contamination in the RNA samples. Additionally, the RNA Integrity Number (RIN) values were ≥7.0, meeting the requirements for library construction, with all ten samples having RIN values greater than 9.0, indicating that the RNA extracted from the samples was of sufficient quality for subsequent analyses.

### 3.10. Sequencing Data Quality Control Analysis

Quality control analysis was performed on the sequencing data generated. As indicated in Table 3, after rigorous data processing steps, including the removal of adapter sequences and low-quality data, clean reads were obtained. The analysis revealed that the Q30 values for all samples were above 97%, signifying that at least 97% of bases were of high accuracy in the sequencing data. Additionally, the GC content ranged from 43.56% to 46.25%, indicating a relatively uniform distribution of bases without significant bias. These results further validate the accuracy and reliability of the sequencing process.

### 3.11. Reference Sequence Alignment

After deduplication, the clean reads were aligned with the reference genome of *C. elegans* (assembly WBcel235). The alignment yielded the following results: the number of reads that mapped to the reference genome (Mapped Reads, MR), the number of reads that mapped uniquely (Unique Mapped Reads, UMR), the number of reads that mapped to the positive strand (Reads Mapped to ‘+’, RM to ‘+’), and the number of reads that mapped to the negative strand (Reads Mapped to ‘−’, RM to ‘−’). As shown in Table 4, the alignment rate for all samples exceeded 98%, and the proportion of reads that mapped uniquely was higher than 90%. Additionally, the distribution of reads mapped to the positive and negative strands of the reference genome was about 50% each, indicating high efficiency and accuracy of the alignment, thus confirming the reliability and accuracy of the sequencing results.

### 3.12. Differential Gene Expression Analysis

**Figure 5:**
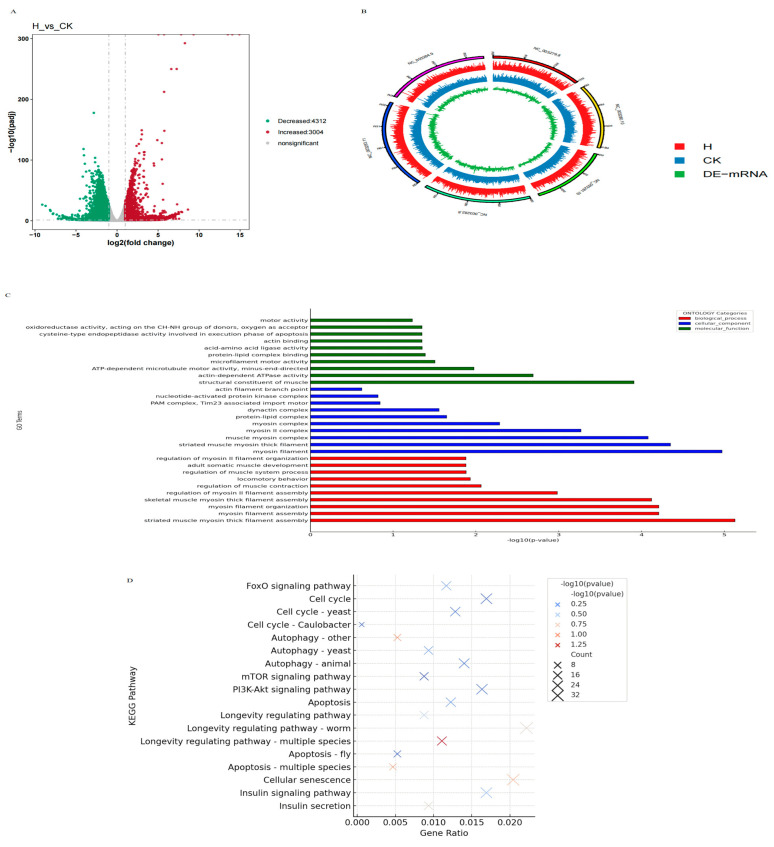
Volcano Plot of Differentially Expressed Genes (**A**); Differential Gene Expression Circos Plot (**B**); GO enrichment analysis of DEGs (**C**); KEGG enrichment analysis of DEGs (**D**). Panel A shows the volcano plot highlighting the distribution of differentially expressed genes (DEGs), where upregulated genes are marked in red and downregulated genes in green. Panel B presents the Circos plot of chromosomal distribution of DEGs, with red indicating upregulated genes and blue indicating downregulated genes, reflecting biologically relevant gene expression states. Panel C displays the GO enrichment analysis results, categorizing DEGs into biological processes (BP), cellular components (CC), and molecular functions (MF), while Panel D shows the KEGG enrichment analysis results, highlighting significant pathways related to metabolism and signaling. In Panels C and D, the color intensities represent enrichment significance, with thresholds set at *p*-value < 0.05 and FDR < 0.05, where darker colors indicate higher significance. A legend has been added to clarify GO terms and KEGG pathways, ensuring accessibility for readers unfamiliar with these terminologies.

Quantitative analysis of transcript expression levels was conducted using the RSEM expression quantification software (version 1.3.3). Differential expression genes were identified based on a fold change (FC) ≥ 1.0 and a *p*-value less than 0.05 between the control group and the blueberry anthocyanin-treated group. Figure 5B displays a genomic circle diagram illustrating the distribution of these differentially expressed genes.

Statistical analysis revealed that compared to the control group, the blueberry anthocyanin-treated group exhibited a total of 7316 significantly differentially expressed genes, with 3004 genes significantly upregulated and 4312 genes significantly downregulated. These differentially expressed genes are primarily involved in biological processes such as growth, metabolism, aging, and stress resistance in nematodes, suggesting that BA may exert their biological effects by regulating these genes. This comprehensive profiling offers insights into the molecular mechanisms through which BA may influence aging and related phenotypes in nematodes.

### 3.13. Differential Gene Expression GO Enrichment Analysis

The GO enrichment analysis of differentially expressed genes in *C. elegans* treated with BA was conducted using Goatools (https://github.com/tanghaibao/goatools), adhering to stringent statistical standards where a *p*-value of less than 0.05 indicates significant enrichment. The analysis revealed significant enrichments in several biological pathways and molecular functions, such as striated muscle myosin thick filament assembly, regulation of myosin II filament assembly, locomotory behavior, and components of the PAM complex associated with the *Tim23* import motor. Furthermore, enhancements were observed in nucleotide-activated protein kinase complexes, actin filament branch points, protein-lipid complex binding, cysteine-type endopeptidase activity involved in apoptosis, and oxidoreductase activities. These findings suggest that BA may influence the aging process by enhancing muscle tissue maintenance and cellular structural integrity, thus providing a scientific basis for further exploration of these compounds as potential anti-aging therapeutic agents.

### 3.14. Differential Gene Expression KEGG Pathway Enrichment Analysis

To systematically understand the molecular mechanisms by which BA extend lifespan, KEGG pathway enrichment analysis was performed to identify the main metabolic and signal transduction pathways involved by differentially expressed genes. As depicted, the top 20 enriched signaling pathways in the blueberry anthocyanin-treated group were identified, with the most significant enrichment observed in pathways regulating *C. elegans* lifespan. This indicates that BA significantly impact nematode longevity, consistent with previous research suggesting that these compounds can extend lifespan by activating the oxidative-reduction balance system.

Additionally, differentially expressed genes (DEGs) were significantly enriched in pathways such as autophagy regulation, the FOXO signaling pathway, the insulin/insulin-like growth factor (IIS) signaling pathway, and the PI3K/Akt signaling pathway. In the FOXO pathway, BA significantly activated *daf-16* (FOXO transcription factor), which regulates stress resistance and lifespan extension by enhancing antioxidant defenses and cellular repair mechanisms. In the IIS pathway, upregulation of *ins-1* (insulin-like peptide), *age-1* (phosphatidylinositol-3 kinase), and *daf-2* (insulin/IGF-1 receptor) was observed, promoting energy metabolism and lifespan regulation. Within the PI3K/Akt pathway, *akt-1* and *akt-2* were significantly modulated, supporting cell growth, survival, and proliferation. These gene-to-function relationships highlight that BA exerts its anti-aging effects by activating stress resistance, energy balance, and cellular survival pathways in *C. elegans*.

These findings demonstrate that BA extend organism lifespan by influencing multiple key signaling pathways, coordinating cellular metabolism, and stress responses. This holistic effect on key pathways elucidates the potential of BA as effective anti-aging compounds.

### 3.15. Validation of Differentially Expressed Genes by Real-Time PCR

**Figure 6:**
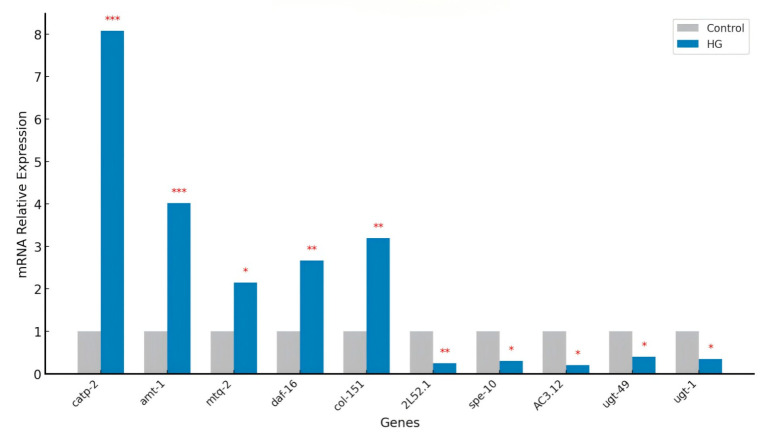
Real-time PCR validation of DEGs from RNA-seq. mRNA relative expression levels were normalized to the reference gene act-1 and are presented as fold changes relative to the control group. Error bars represent the standard error of the mean (SE) calculated from three independent biological replicates. Statistical significance is indicated by asterisks (* *p* < 0.05, ** *p* < 0.01, *** *p* < 0.001).

In this study, as shown in Figure 6 the transcriptomic sequencing data for nematodes treated with BA showed that the expression levels of key genes such as *catp-2*, *amt-1*, *mtq-2*, *daf-16*, and *col-151* were upregulated by 3.05, 2.02, 1.09, 1.42, and 1.67 times, respectively. Further validation through real-time PCR analysis confirmed these results, showing that the expression levels of *catp-2*, *amt-1*, *mtq-2*, *daf-16*, and *col-151* in the blueberry anthocyanin-treated group were 8.08, 4.02, 2.15, 2.67, and 3.20 times higher than in the control group, respectively.

## 4. Discussion

This study explored the potential anti-aging effects of blueberry anthocyanins (BA) on Caenorhabditis elegans, a widely used model organism in aging research [26,27]. Through measuring various phenotypic indicators, we found that BA significantly extended the lifespan of *C. elegans* and improved their locomotor abilities, pharyngeal pumping efficiency, reproductive capacity, and antioxidant defenses [28]. Anthocyanins, as polyphenolic compounds, are widely recognized for their dual roles: as direct ROS scavengers and as regulators of cellular signaling pathways. While their low intracellular concentrations may limit their direct antioxidant activity in larger organisms, their high water-solubility could enhance bioavailability in smaller organisms such as *C. elegans*, enabling some degree of ROS scavenging. On the other hand, this solubility may reduce their affinity for regulatory protein active sites, tilting their function toward direct antioxidants rather than signaling regulators. Nevertheless, our study clearly demonstrates that BA significantly impacts key aging-related signaling pathways, such as FOXO and IIS, which are essential for stress resistance, energy metabolism, and lifespan regulation. This suggests that BA may exert its anti-aging effects through both direct antioxidant activity and indirect regulation of cellular pathways. Future studies should focus on quantifying ROS levels and assessing the balance between BA’s direct and indirect effects, which will provide a more comprehensive understanding of its mechanisms and potential applications as a therapeutic agent against aging and age-related diseases.

First, the lifespan extension experiment showed that BA had a dose-dependent effect on the lifespan of *C. elegans*. All three concentrations tested (50.0, 200.0, and 500.0 μg/mL) led to an increase in lifespan, with the highest concentration (500.0 μg/mL) showing the most pronounced effect. This finding is consistent with existing literature indicating that dietary interventions, including plant-derived compounds, can modulate the aging process in model organisms. For instance, Zhang [29] reported that anthocyanin extracts significantly prolonged the lifespan of *C. elegans* by enhancing stress resistance and modulating oxidative stress pathways. Similarly, Xu [30] observed that plant-derived polyphenols enhanced longevity by regulating key signaling pathways like IIS and FOXO. The relationship between BA concentration and lifespan extension highlights the importance of dosage optimization for potential anti-aging therapies. Moreover, the results demonstrate that aging is not an irreversible process and that external factors such as diet and bioactive compounds can modulate aging and delay age-related decline.

Second, we investigated the effects of BA on *C. elegans*’ locomotor ability, an important indicator of muscle function and overall health. The results showed that BA treatment significantly improved the bending frequency of *C. elegans* [31], especially at higher concentrations (200.0 and 500.0 μg/mL), though no significant difference was observed between these two concentrations. This suggests that BA may alleviate the decline in locomotor function associated with aging by targeting muscle maintenance or related pathways. Previous studies also support this conclusion: Yan [32] demonstrated that plant polyphenols like resveratrol could improve motor performance in *C. elegans* by enhancing mitochondrial function and reducing oxidative stress. Moreover, Li [33] found that antioxidants derived from anthocyanins mitigated age-related muscle deterioration by activating stress response pathways. These findings, combined with our results, indicate that BA-treated nematodes show significantly enhanced motor performance compared to the control, demonstrating the potential of BA in preserving locomotor ability during aging.

Additionally, we evaluated the impact of BA on other physiological processes, such as pharyngeal pumping efficiency and reproductive health. Pharyngeal pumping rate is a crucial indicator of feeding efficiency and metabolic health [34,35]. The results showed that BA treatment significantly improved the pharyngeal pumping rate, suggesting that BA not only promotes food intake but also supports overall health, which may contribute to lifespan extension. Given the strong association between food intake, metabolism, and longevity, this enhancement in pharyngeal pumping likely aids in maintaining health during aging by ensuring optimal nutrient uptake [36]. Furthermore, BA-treated nematodes showed enhanced reproductive output, suggesting that BA may help delay reproductive aging. Reproductive health is a key indicator of aging in many organisms, which further underscores the potential of BA as a multifaceted anti-aging agent [37].

Oxidative stress is a significant factor in aging, driven by the accumulation of reactive oxygen species (ROS) and substances like lipofuscin [38,39]. In this study, we observed that BA treatment significantly reduced lipofuscin accumulation in aging *C. elegans*, and the fluorescence intensity decreased progressively with increasing BA concentration, suggesting that BA may mitigate oxidative damage by reducing lipofuscin levels [40,41]. This was supported by the observed reduction in ROS levels in BA-treated nematodes, detected using the DCFH-DA fluorescent probe. By reducing ROS accumulation and oxidative damage, BA may delay the decline in cellular function associated with aging. These findings indicate that the anti-aging effects of BA are largely attributable to their antioxidant properties, consistent with other studies showing that antioxidants can extend lifespan by alleviating oxidative stress [42,43].

We also performed transcriptomic analysis to understand the molecular mechanisms underlying the anti-aging effects of BA. Quality control of the RNA used for sequencing was a critical aspect of ensuring the reliability of the transcriptomic data [44]. All RNA samples used for sequencing exhibited OD 260/280 ratios above 1.80, which confirmed that there was no significant contamination with proteins or other impurities [45]. The OD 260/230 ratios ranged from 2.1 to 2.6, indicating the absence of organic reagent contamination [46]. Moreover, the RNA Integrity Number (RIN) values were all ≥7.0, with all samples achieving RIN values greater than 9.0, demonstrating that the RNA was of high quality and suitable for sequencing. After rigorous quality control steps—including removal of adapters and low-quality sequences—the sequencing data showed a Q30 value exceeding 97% for all samples, indicating a high level of sequencing accuracy. The GC content ranged from 43.56% to 46.25%, signifying a relatively uniform base distribution without significant bias, further validating the reliability of the data [47]. These stringent quality control measures ensure that the subsequent analysis of differential gene expression and pathway enrichment is based on highly reliable data [48].

Transcriptomic analysis revealed important insights into the potential molecular mechanisms of BA-induced lifespan extension [49]. One of the most significant findings was the activation of the FOXO signaling pathway, particularly through the upregulation of the *daf-16* transcription factor, which is known to regulate stress responses and longevity in *C. elegans* [50]. The bioactive anthocyanin compounds in blueberry anthocyanins (BA), such as Cyanidin-3-glucoside, Delphinidin-3-glucoside, Petunidin-3-glucoside, Malvidin-3-glucoside, and Peonidin-3-glucoside, may exert their anti-aging effects through the modulation of key aging-related signaling pathways in Caenorhabditis elegans [51,52,53]. These pathways include the insulin/insulin-like growth factor (IIS) and PI3K/Akt pathways, which are critical regulators of lifespan and stress resistance. BA may suppress IIS components such as daf-2 and age-1, reducing phosphorylation of the FOXO transcription factor *daf-16* and promoting its nuclear translocation. Once activated, *daf-16* upregulates genes involved in stress resistance and longevity. Furthermore, BA may inhibit the PI3K/Akt pathway, similarly allowing for the activation of *daf-16*. These mechanisms suggest that BA compounds exert anti-aging effects via a dual role: acting as direct ROS scavengers due to their high water solubility and as regulators of cellular signaling pathways, ultimately contributing to stress resistance, improved metabolism, and extended lifespan in *C. elegans*. The upregulation of *daf-16* suggests that BA enhances the nematodes’ resistance to environmental stress. Consistent with previous studies, the activation of *daf-16* has been shown to play a critical role in metabolic remodeling and stress tolerance. For example, Zečić and Braeckman [54] demonstrated that *daf-16* promotes mitochondrial function and energy metabolism through the regulation of key metabolic genes, which contributes to lifespan extension in *C. elegans*. Similarly, Dilberger [55] found that *daf-16* activation facilitates metabolic optimization, particularly in pathways linked to mitochondrial energy production and protein synthesis, further supporting its central role in aging regulation.

In this study, BA treatment significantly upregulated genes involved in mitochondrial function and energy metabolism, such as *catp-2* and *amt-1*, suggesting that BA may extend lifespan by enhancing energy production and protein synthesis. These molecular changes imply that BA affects a variety of biological pathways, collectively contributing to lifespan extension by improving mitochondrial function, activating key transcription factors related to longevity, and optimizing metabolic processes. Additionally, the observed downregulation of detoxification and cellular stress response genes, such as *ugt-49* and *ugt-1*, may conserve energy and mitigate metabolic stress, further supporting the role of BA in promoting longevity through multi-faceted mechanisms.

In conclusion, this study presents compelling evidence that BA can extend the lifespan of *C. elegans* through multiple biological pathways. By reducing oxidative stress, enhancing energy metabolism, and improving stress responses, BA exerts a comprehensive effect on aging, promoting healthier aging phenotypes. The activation of key signaling pathways, such as FOXO and PI3K/Akt, as well as improvements in mitochondrial function and antioxidant defenses, underscore the potential of BA as effective anti-aging compounds. The stringent quality control of RNA and sequencing data ensures that the findings are reliable, providing a robust foundation for understanding the molecular basis of BA’s anti-aging effects. These findings not only deepen our understanding of BA’s anti-aging mechanisms but also provide a scientific basis for exploring their potential as therapeutic agents for age-related diseases. Future research should further investigate the efficacy of these compounds in higher organisms and elucidate their molecular targets to determine their potential for treating aging-related disorders in humans.

## 5. Conclusions

This study presents compelling evidence that blueberry anthocyanins (BA) can extend the lifespan of Caenorhabditis elegans through multiple biological pathways. Aging is primarily driven by external environmental stresses and genetic factors, and BA, known for their antioxidant properties, effectively mitigate these effects. Using *C. elegans* as a model organism, we demonstrated that BA treatments significantly extended lifespans in a dose-dependent manner (1.15, 1.24, and 1.36 times, respectively), while also improving mobility and pharyngeal pumping abilities. These treatments reduced lipofuscin and ROS accumulation, suggesting mitigation of oxidative damage. Transcriptomic analyses further revealed that BA influence longevity by modulating gene expressions related to muscle function, stress resistance, autophagy, and the FOXO signaling pathway, notably upregulating crucial anti-aging genes such as *daf-16*. Moreover, BA exert their anti-aging effects by reducing oxidative stress, enhancing energy metabolism, and improving stress responses, which collectively promote healthier aging phenotypes. The activation of key signaling pathways, such as FOXO and PI3K/Akt, along with improvements in mitochondrial function and antioxidant defenses, underscores the potential of BA as effective anti-aging compounds. The stringent quality control of RNA and sequencing data ensures that these findings are reliable, providing a robust foundation for understanding the molecular basis of BA’s anti-aging effects. These results not only deepen our understanding of BA’s mechanisms but also provide a scientific basis for exploring their potential as therapeutic agents for age-related diseases. Future research should further investigate the efficacy of these compounds in higher organisms and elucidate their molecular targets to determine their potential for treating aging-related disorders in humans.

## Figures and Tables

**Table 1:** Primer sequences used for real-time PCR.

Gene	Primer
catp-2	F: AGGGACATCAATAGATCCCCTC R: CACGACGTGATAGCCGATGA
amt-1	F: CTCACTCACTGGGGTTGGAC R: GCGTCAAGAGGATCGTCGAT
mtq-2	F: CAGTATCGGCTCCAAGCGAT R: TGCTCCGAGTGCTCTTTGAG
*daf-16*	F: GCGGAGCCAAGAAGAGGA R: CGATTGAGTTCGGGGACG
col-151	F: CCAATGGGACCACAAGGACA R: ACTCGTCTCCAGTCCGAAGA
2L52.1	F: GGTCGGAGGAAGTCGTTTGT R: GGCCGACTTTCCTGTTGAGA
spe-10	F: TATCAAACCGGATCGTGCCC R: AATCGATTCCGTTGCTGGGT
AC3.12	F: GCAGTGGTCTAACATGCTATGC R: TCGCAATACTTCATTCCAGTCA
ugt-49	F: TCGACGTGGCATTCAGTGAA R: CTGGATTGCACGCAATACCC
ugt-1	F: CGAACTCGCATTCTCGAAGC R: AAAGTACTGCCGAGACCACC

**Table 2:** Quality control results of RNA-seq.

Samples	Volume/μL	OD260 nm/280 nm	OD260 nm/230 nm	RIN
ck1	100	1.92	2.49	9.37
ck2	100	1.92	2.52	9.56
ck3	100	1.93	2.13	9.35
ck4	100	1.92	2.46	9.27
ck5	100	1.91	2.32	9.58
H1	100	1.89	2.45	9.67
H2	100	1.89	2.49	9.60
H3	100	1.89	2.52	9.22
H4	100	1.88	2.17	9.12
H5	100	1.88	2.33	9.89

Samples beginning with “CK” are part of the control group (0 μg/mL) and include five replicates. Samples starting with “H” are from the treatment group, which received 500 μg/mL of BA, also comprising five replicates. This labeling ensures clear differentiation between the control and treated groups, facilitating accurate data analysis and comparison across the experimental conditions.

**Table 3:** Quality control results of sequencing data.

Samples	Clean Reads	Clean Bases	GC_Content/%	Q30/%
ck1	49,475,438	7,280,696,512	43.56	97.93
ck2	50,933,490	7,491,886,548	44.07	97.83
ck3	37,923,918	5,586,061,770	44.93	97.87
ck4	45,299,786	6,665,289,633	44.90	97.79
ck5	49,686,856	7,306,834,708	44.38	97.87
H1	52,282,188	7,680,398,938	46.25	97.66
H2	50,246,868	7,382,451,016	45.86	97.73
H3	41,226,402	6,051,907,843	45.79	97.60
H4	47,449,044	6,968,049,163	46.20	97.58
H5	42,691,498	6,236,987,977	45.40	97.38

The table parameters for sequencing data quality control are defined as follows: Samples: The name of the sample. Clean reads: The total number of pair-end reads in the clean data. Clean bases: The total number of bases in the clean data. GC content: The percentage of guanine (G) and cytosine (C) bases in the clean data. Q30: The percentage of bases in the clean data with a quality score of 30 or higher.

**Table 4:** Statistical comparison between sequencing data and reference genome sequences.

Sample	Total Reads	MR	UMR	RM to‘+’	RM to‘−’
ck1	49,475,438	98.51%	93.82%	50	50
ck2	50,933,490	98.58%	94.45%	50	50
ck3	37,923,918	98.71%	95.12%	50	50
ck4	45,299,786	98.71%	95.15%	50	50
ck5	49,686,856	98.54%	94.06%	50	50
H1	52,282,188	98.31%	90.90%	50	50
H2	50,246,868	98.50%	92.88%	50	50
H3	41,226,402	98.40%	92.73%	50	50
H4	47,449,044	98.34%	91.70%	50	50
H5	42,691,498	98.55%	92.62%	50	50

## Data Availability

The original contributions presented in the study are included in the article, further inquiries can be directed to the corresponding authors.
